# Improving How Caregivers of People Living With Dementia Are Identified in the Electronic Health Record: Qualitative Study and Exploratory Chart Review

**DOI:** 10.2196/59584

**Published:** 2024-12-13

**Authors:** Ariel R Green, Cynthia M Boyd, Rosalphie Quiles Rosado, Andrea E Daddato, Kathy S Gleason, Tobie E Taylor McPhail, Marcela D Blinka, Nancy L Schoenborn, Jennifer L Wolff, Elizabeth A Bayliss, Rebecca S Boxer

**Affiliations:** 1 Division of Geriatric Medicine and Gerontology, School of Medicine, Johns Hopkins University, Mason F Lord Building, Center Tower, 5200 Eastern Avenue, 7th Floor, Baltimore, MD, 21224, United States, 1 410 550 6733; 2 Institute for Health Research, Kaiser Permanente Colorado, Aurora, CO, United States; 3 Department of Health Policy and Management, Bloomberg School of Public Health, Johns Hopkins University, Baltimore, MD, United States; 4 Department of Medicine, University of California, Davis, Sacramento, CA, United States

**Keywords:** dementia, dementia care, caregivers, electronic health record, patient record, aging, geriatrics, memory

## Abstract

**Background:**

Family and unpaid caregivers play a crucial role in supporting people living with dementia; yet, they are not systematically identified and documented by health systems.

**Objective:**

The aims of the study are to determine the extent to which caregivers are currently identified and documented in the electronic health record (EHR) and to elicit the perspectives of caregivers and clinical staff on how to best identify, engage, and support caregivers of people living with dementia through the EHR.

**Methods:**

People with dementia were identified based on *International Classification of Diseases, Tenth Revision* (*ICD-10*) codes or dementia medications in the EHR. A chart review of people with dementia characterized how caregiver information was documented and whether caregivers had shared access to the patient portal. Caregivers of eligible people with dementia were then recruited through mailed letters and follow-up calls to the homes of people with dementia. We conducted semistructured interviews with caregivers, clinicians, and staff involved in the care of people with dementia within 2 health systems in Maryland and Colorado. Transcripts were analyzed using a mixed inductive and deductive approach.

**Results:**

Caregivers of people with dementia (N=22) were usually identified in the “contact information” or “patient contacts” tab (n=20, 91%) by their name and relation to the people with dementia; this tab did not specify the caregiver’s role. Caregivers were also mentioned, and their roles were described to a varying degree in clinical notes (n=21, 96%). Of the 22 caregivers interviewed, the majority (n=17, 77%) reported that the people with dementia had additional caregivers. The presence of multiple caregivers could be gleaned from most charts (n=16, 73%); however, this information was not captured systematically, and caregivers’ individual contributions were not explicitly recorded. Interviews with 22 caregivers and 16 clinical staff revealed two major themes: (1) caregiving arrangements are complex and not systematically captured or easy to locate in the EHR and (2) health systems should develop standardized processes to obtain and document caregiver information in the EHR.

**Conclusions:**

This exploratory chart review and qualitative interview study found that people with dementia frequently have multiple caregivers, whose roles and needs are captured inconsistently in the EHR. To address this concern, caregivers and clinical staff suggested that health systems should develop and test workflows to identify caregivers, assess their needs at multiple touchpoints, and record their information in extractable EHR fields.

## Introduction

The engagement of family and unpaid caregivers is critical for improving care for people living with dementia [1]. More than 11 million family members and other unpaid caregivers provide an estimated 18 billion hours of care to people living with dementia [2]. Caregivers assist with daily activities, cope with behavioral symptoms, schedule and attend medical visits, manage medications, and make medical decisions [3]. Health systems lack systematic and proactive processes to identify and engage with caregivers, meaning that they remain largely invisible [4,5].

The electronic health record (EHR) is a promising tool to enable timely, accurate, bidirectional information exchange and communication between caregivers and health care teams through applications such as the patient portal. Despite the nearly universal adoption of the EHR [6], its application to dementia care remains limited. Developing a feasible approach to identify caregivers of people living with dementia in the EHR would enable health systems to reach them equitably and at scale, include them in decisions, provide support, and recruit them for research that would benefit them [7]. As little is known about how best to achieve this, we conducted an exploratory chart review and qualitative study with caregivers and clinical staff to characterize how caregivers are currently identified and documented in the EHR in 2 health care systems and elicit perspectives on how best to identify, engage, and support caregivers of people living with dementia through the EHR. Qualitative methods are ideal for this purpose, as they can provide a deep understanding of caregivers’ lived experiences and of caregiver and staff interactions with the EHR and generate hypotheses about how health systems can better use the EHR as a tool to support caregivers.

## Methods

### Study Design

This analysis was done as part of a study designed to develop a tool to identify caregivers of people living with dementia through the EHR for pragmatic trials related to medication management. Caregivers of people living with dementia and clinical staff from 2 health systems in Maryland and Colorado were recruited from March to May 2022. Research staff screened the EHR to identify patients aged 65 years or older with dementia (based on *International Classification of Diseases, Tenth Revision* [*ICD-10*] codes or dementia medications; Multimedia Appendix 1) and polypharmacy (defined as taking 5+ medications), who received care at primary care or memory clinics. The dementia diagnosis was confirmed by reviewing clinic notes. Our approach was designed to be pragmatic to characterize the real-world caregiving arrangements of people with dementia. For this reason, we sought to include caregivers regardless of how much time they spent caring for the people living with dementia. To maximize generalizability, we used broad eligibility criteria for caregivers and allowed them to self-identify. Recruitment letters addressed to “family members or friends” were mailed to the homes of eligible people living with dementia. We then called the people living with dementia to identify a caregiver interested in participating. Staff who provided ambulatory care to people living with dementia were identified through lists provided by clinics and recommendations of clinic administrators. They were recruited by email. We stopped recruitment once no new themes were emerging in interviews.

Chart review of people living with dementia whose caregivers participated focused on characterizing whether caregiver identities, contact information, and roles were documented in the EHR (and if so, where) and whether caregivers had registered for proxy access to the patient portal. We developed a chart abstraction form in Microsoft Excel. Two research coordinators (RQR and AED) reviewed charts in the EHR and recorded their findings. These data were discussed with the principal investigator (ARG) during team meetings to identify and adjudicate discrepancies. We conducted semistructured interviews with 22 caregivers and 16 clinical staff (eg, clinicians, nurses, medical assistants, and practice managers). An interview guide was developed based on the research questions. We asked caregivers about their roles and caregiving arrangements and compared this with the information that was documented in the EHR. We also asked whether the patient’s health care team ever routinely asked them about caregiving roles and responsibilities and, if so, when. We asked staff how they currently identify caregivers and record their information. Both groups were asked about challenges related to this process and how it could be improved. All participants were also asked for demographic information. Four research team members (RQR, AED, KSG, and TETM) experienced in qualitative research conducted the interviews via phone or videoconference; all interviews were conducted in private locations. Two coders (KSG and TETM) developed an initial codebook based on the Consolidated Framework for Implementation Research and the study aims. Three study team members then refined the codebook by reading transcripts from 5 caregivers and 5 staff. Open coding allowed inductive identification of new themes in addition to deductive coding guided by the Consolidated Framework for Implementation Research. The entire team met to discuss and finalize the codebook. Using this version, a single team member coded approximately 25% (n=8) of the remaining transcripts, which were reviewed by another coder. Discrepancies were discussed at study team meetings until 100% agreement was reached. The remaining transcripts were then coded by a single coder, and the study team met weekly to discuss emerging themes.

### Ethical Considerations

The Johns Hopkins University School of Medicine Institutional Review Board (IRB) served as the single IRB and approved this research (IRB protocol 00297952); the Kaiser Permanente Colorado IRB ceded. All participants provided verbal consent. Caregivers at both health systems and clinical staff in Maryland received a US $25 gift card for participating; clinical staff in Colorado volunteered without compensation, as company policy did not allow it. All interviews were audio recorded and transcribed verbatim.

## Results

### Overview

Participant characteristics are described in Table 1. People with dementia (n=22) had a mean age of 84.1 (SD 6.5) years. Most were male (n=15, 68%) and White (n=15, 68%) and had a mean of 8 (SD 3.5) medications.

**Table 1.** Demographic characteristics of participants.

	People with dementia^a^(n=22)	Caregiver^b^(n=22)	Staff^b^(n=16)
Age (years), mean (SD)	84.1 (6.5)	63 (12.6)	48 (9.3)^c^
**Sex, n (%)**			
Female	7 (32)	21 (96)	12 (75)
Male	15 (68)	1 (5)	2 (13)
Other or unknown	0 (0)	0 (0)	2 (13)
**Race, n (%)**			
Asian	0 (0)	0 (0)	3 (19)
Black or African American	5 (23)	5 (23)	0 (0)
White	15 (68)	16 (73)	9 (56)
Other	1 (4.5)	1 (5)	1 (6)
Unknown or not reported	1 (5)	0 (0)	3 (19)
**Ethnicity, n (%)**			
Hispanic or Latino	1 (5)	1 (5)	1 (6)
Not Hispanic or Latino	20 (91)	21 (96)	15 (94)
Unknown or not reported	1 (5)	—^d^	—
Total number of medications, mean (SD)	8 (4)	—	—
**Confidence filling out medical forms, n (%)**	—		—
Extremely		16 (73)	
Quite a bit		5 (23)	
Somewhat		1 (5)	
A little bit		0 (0)	
Not at all		0 (0)	
**Relationship to people with dementia** ^c^ **, n (%)**	—		—
Spouse or partner		9 (41)	
Adult child		9 (41)	
Other (friend or other relative)		4 (18)	
Lives with the people with dementia, n (%)	—	14 (64)	—
**Occupation, n (%)**	—	—	
Physician			4 (25)
Physician assistant			2 (13)
Medical assistant			2 (13)
Nurse (LPN^e^, RN^f^, NP^g^)			6 (38)
Practice manager			2 (13)
**People with dementia identified via, n (%)**		—	—
Dementia diagnosis on problem list	19 (86)		
Dementia medication on medication list	10 (46)		

a Data based on chart review.

b Data based on interviews.

c Three staff members did not provide their ages.

d These questions were not asked of both groups.

e LPN: licensed practical nurse.

f RN: registered nurse.

g NP: nurse practitioner.

Caregivers had a mean age of 63 (SD 12.6) years. The majority were female (n=21, 96%) and White (n=16, 73%). Most were the spouse, partner, or adult child of the people with dementia (n=18,82%) and lived with the people with dementia (n=14, 64%). Only 1 caregiver had proxy access to the patient portal. As shown in Table 2, caregiver names and their relation to the people with dementia were usually recorded in the “contact information” or “patient contacts” tab of the chart (n=20, 91%). Caregivers were frequently mentioned in clinical notes (n=21, 96%), where their relations and roles were described with a varying degree of detail (eg, patient lives independently and son helps). One of the health systems had a “lay caregiver” field that was used inconsistently (n=3, 27%). Most people with dementia (n=13, 59%) had an advance directive in the chart that named a medical decision maker. When an advance directive was present, it identified the same caregiver as interviewed only slightly more than half of the time (n=12, 55%). Of the 22 caregivers interviewed, the majority (n=17, 77%) reported that the people with dementia had additional caregivers. Most charts (n=16, 73%) contained documentation implying the presence of multiple caregivers; this information was recorded unsystematically in various locations throughout the chart, and caregivers’ individual contributions were not explicitly described. Two major themes emerged from interviews (described with illustrative quotations in Table 3).

**Table 2.** Results of chart review and interviews related to caregiver identification in the EHR^a^ (N=22).

Category	Values, n (%)
Interviewed caregiver identified multiple caregivers^b^	17 (77)
Chart review identified multiple caregivers	16 (73)
**Locations where caregivers were identified^c^ in the EHR**	
Contact information^d^	20 (91)
Clinical notes	21 (96)
“Lay caregiver” field	3 (27)^e^
**People with dementia had patient portal access**	
Yes	18 (82)
No	1 (5)
Inactive	3 (14)
**Caregiver had proxy access to the patient portal**	
Yes	1 (5)
No	19 (86)
Unknown (patient deceased)	2 (9)
**People with dementia had an advance directive (ie, living will or medical durable power of attorney) in the chart that named a medical decision maker**	
Yes	13 (59)
No	7 (32)
Unknown (patient reported having advance directive but copy not in chart)	2 (9)
**If an advance directive was present, it identified the same caregiver as interviewed**	
Yes	12 (55)
No	1 (5)
Unknown (patient reported having advance directive but copy not in chart)	2 (9)

a EHR: electronic health record.

b Data obtained from the interview; all other data in the table were obtained from chart review.

c Fields were not mutually exclusive.

d Caregivers were identified in various fields within the “Demographics” or “Snapshot” tabs, depending on the health system, such as “permanent comments,” “patient contacts,” “additional info,” and “alternate contact person.”

e This field only existed at 1 health system; denominator=11.

**Table 3.** Key themes and illustrative quotations.

Participant	Quote
**Theme 1: complexity of caregiving arrangements not systematically captured or easy to locate in the EHR^a^**	
Caregiver	“We’ll spread it out...I am the one that goes to the appointments. If...she needs to be on something new, I work with the doctor and then talk to my sister about it.... I generally will have the current list or whoever has been at the last few doctors’ appointments.”
Medical assistant	“[The problem list] shows dementia...but there’s not any real information in regard to who the caregiver is.”
Nurse case manager	“It’s a lot of time devoted to...detective work – trying to peel the layers back to figure out who would be an appropriate person to truly get good information from.”
Physician	“We don’t have a system.... So I do it my way. Another doctor does it their way. A third person does it their way.... In my initial new patient assessment, I put it in my note, but you need to know where to find it.”
**Theme 2: health systems should develop standardized processes to obtain and document caregiver information in the EHR**	
Caregiver	“No one seemed to know what the correct procedure was [to register for shared access in the patient portal].... I was told to fill out this form. Later I found out ‘No, that was not the form you were supposed to fill-out.’ ...I drove over to the medical clinic 45 minutes each way to get this done.”
Caregiver	“I’d like, somehow, when [clinicians] sign into [the EHR] that it’s apparent that she has dementia and that her daughter-in-law is the caregiver and that there’s a banner there right away so I don’t always have to explain myself.... It’s necessary [that] I be with her because she is incapable of giving them the information they need.... Somehow, in My Chart, that information would be...a quick blip on the page.”
Caregiver	“Every now and then, just ask me how I’m doing.... It’s kind of hard to answer with him sitting there.”
Nurse case manager	“[The EHR should include] what services they provide, [do] they grocery shop for [the PLWD], prepare meals for them, provide transportation, medication management – all the big things that you’re concerned with a memory-impaired person – who can be relied on to help.”
Clinic medical director	“I think it would be helpful if [the caregiver information] was a banner that went across [the EHR], so that it’s highlighted. So, if a patient has an active health care agent right now, when you open the chart, it’s a banner, and it has the name and the number listed. And every time you go within that patient’s chart, even every single note that you bring up, has that banner.… But, otherwise, everything else would get lost, the information that was collected.”
Physician	“If we can also add [caregiver information] into Epic [so] that we don’t need to dig...the problem is you need to know it and you always need to look. There [are] so many scanned papers, but if they can add...the contact information [to a] box...[the] primary caregiver and...the other caregivers, that would be useful.”

a EHR: electronic health record.

### Complexity of Caregiving Arrangements Not Systematically Captured or Easy to Locate

Interviews revealed that most people living with dementia had multiple people who helped them, including family, friends, and community members. Some people who provided instrumental support did not view themselves as caregivers, complicating health system efforts to identify them. Caregivers said that their needs (eg, skills or resources needed for caregiving) were rarely assessed or recorded by the health system. Staff said caregiver information was not collected systematically and that caregiver roles, such as which caregiver helps with medical decision-making and daily activities, including medication administration, were usually described in clinical notes rather than readily accessible fields. This often made it time-consuming and difficult to identify the appropriate caregiver to contact for information exchange. Even when a caregiver was identified by name or relation to the patient, it was often not easy to determine if that caregiver was the relevant person for a particular clinical decision, such as a medication change.

### Health Systems Should Develop Standardized Processes to Obtain and Document Caregiver Information

Caregivers expressed a desire to be identified and have their needs routinely assessed and addressed. Clinical staff recommended developing workflows to identify caregivers and assess their needs at multiple touchpoints, including standardized questions in the waiting room, the patient portal, and during scheduling. Staff said that it would be useful to collect information on what tasks caregivers perform and suggested adding a caregiver tab to the EHR to standardize what information is collected and where it is recorded. Caregivers and staff suggested that proxy access to patient portals could facilitate improved identification, engagement, and support of caregivers. However, the complicated proxy enrollment process, which requires downloading and completing forms and providing proof of legal representative status if the patient lacks decision-making capacity, was seen as a barrier.

## Discussion

### Principal Findings

This exploratory chart review and qualitative study found that people living with dementia frequently have multiple caregivers, whose roles and needs are inconsistently captured in the EHR. Our findings were similar in both health systems. Caregivers emphasized the importance of having their needs routinely evaluated and addressed. Clinical staff suggested creating processes to identify caregivers and assess their needs at various points, such as using standardized questions in the clinic waiting room, through the patient portal, and during appointment scheduling. Participants recommended simplifying the process of obtaining proxy access to patient portals, particularly for caregivers of people living with dementia.

Similar to our findings, a previous study of 211 people living with dementia that did not involve interviews or determine whether people living with dementia had multiple caregivers found that 89% of charts identified caregivers; however, “an extensive search through individual notes” was required to locate caregiver information; few charts documented caregiver needs [8]. These findings have important implications. Caregiver engagement and support are essential components of high-quality dementia care. Patients with poorly supported caregivers face a heightened risk for inappropriate medication use [9], hospitalization, and burdensome treatments [10]. Pragmatic trials may exclude people with dementia when there is no documentation of a caregiver, limiting generalizability [11].

Earlier research has shown that patient portals can be a valuable resource for supporting patients with serious illness and their caregivers [12]. This is illustrated by the fact that patient portal use, such as secure messaging, tends to increase following a dementia diagnosis, reflecting the substantial need for information and communication of people living with dementia and their caregivers [13]. In a pilot study, caregivers of people living with dementia perceived that they received more information about resources from the doctor after completing a caregiver wellness questionnaire in the patient’s EHR [14]. Another study tested an EHR-driven process to identify veterans with unpaid caregivers based on receipt of home- and community-based referrals; however, the process was inefficient [4]. Shared access to the patient portal would facilitate the identification and enable the engagement of multiple caregivers, reflecting the reality of caregiving arrangements for people living with dementia [15]. However, health systems should simplify the complicated proxy enrollment process, which is a barrier [16,17]. To address the concerns identified in our study, health systems should develop and test workflows to identify caregivers, assess their needs at multiple touchpoints, and record their roles and contact information in standardized, clearly defined, and readily extractable fields. These workflows could include fielding caregiver questions as part of the Medicare Annual Wellness Visit, creating a communication plan at the time of dementia diagnosis, and testing different modes of outreach to people living with dementia and caregivers [15,16].

### Limitations

This study has some limitations. The small sample from 2 health care systems may not be generalizable to other practice settings. In addition, caregivers who could be identified during recruitment and who agreed to participate may be more likely to be engaged and identified in the EHR. Our broad inclusion criteria may have resulted in people who only spent a few hours caregiving being classified as caregivers. Although both health systems used the Epic EHR, the tabs and fields were different in each system, and the locations where we found caregiver information may not be generalizable. As a qualitative study, this work is designed to be exploratory. However, it is valuable because it provides an in-depth understanding of caregiving arrangements for people living with dementia and of the lived experiences of dementia caregivers in their interactions with the EHR. In so doing, the study generates important hypotheses about how health systems can better support caregivers. Finally, we did not seek to interview paid caregivers, who may be responsible for the day-to-day care of the people living with dementia and accompany them to medical appointments [18].

### Conclusions

This qualitative study and exploratory chart review found that caregivers of people living with dementia are identified inconsistently in the EHR. If their roles and needs are documented, this information is not easy to locate. Future studies could develop and test workflows to identify caregivers, assess their needs, and record their information in extractable EHR fields.

## Supplementary material

10.2196/59584 Multimedia Appendix 1 *International Classification of Diseases, Tenth Revision* (*ICD-10*) codes and medications used to identify people living with dementia.

## Abbreviations

EHR: electronic health record
*ICD-10*: *International Classification of Diseases, Tenth Revision*
IRB: institutional review board
