# Supplementary material for: Improving How Caregivers of People Living With Dementia Are Identified in the Electronic Health Record: Qualitative Study and Exploratory Chart Review
Source: JMIR Aging. 2024 Dec 13;7:e59584. doi: 10.2196/59584 (PMC11660723; doi:10.2196/59584)
Supplement: Multimedia Appendix 1 [file aging-v7-e59584-s001.docx]

| **ICD** | **Code** | **Category** | **Definition** |
| --- | --- | --- | --- |
| 10 | F01.50 | Dementia | Vascular dementia without behavioral disturbance |
| 10 | F01.51 | Dementia | Vascular dementia with behavioral disturbance |
| 10 | F02.80 | Dementia | Dementia in other diseases classified elsewhere without behavioral disturbance |
| 10 | F02.81 | Dementia | Dementia in other diseases classified elsewhere with behavioral disturbance |
| 10 | F03.90 | Dementia | Unspecified dementia without behavioral disturbance |
| 10 | F03.91 | Dementia | Unspecified dementia with behavioral disturbance |
| 10 | F03.92 | Dementia | Presenile with delusional features |
| 10 | F03.93 | Dementia | Presenile with depressive features |
| 10 | F06.8 |  | Other specified mental disorders due to known physiological condition |
| 10 | F19.97 |  | Other psychoactive substance use, unspecified with psychoactive substance-induced persisting dementia |
| 10 | G30.0 | Alzheimer’s | Alzheimer’s disease with early onset |
| 10 | G30.1 | Alzheimer’s | Alzheimer’s disease with late onset |
| 10 | G30.8 | Alzheimer’s | Other Alzheimer’s disease |
| 10 | G30.9 | Alzheimer’s | Alzheimer’s disease, unspecified |
| 10 | G31.01 | Other | Picks disease |
| 10 | G31.09 | Dementia | Other frontotemporal dementia |
| 10 | G31.1 | Degeneration | Senile degeneration of brain, not elsewhere classified |
| 10 | G31.83 | Dementia | Dementia with Lewy bodies |
| 10 | G31.89 |  | Other specified degenerative diseases of nervous system |
| 10 | G31.9 | Degeneration | Degenerative disease of nervous system, unspecified |
| 10 | R41.3 | Memory Loss | Other amnesia |
| **Medications** | | | |
| Donepezil (Aricept)  Rivastigmine (Exelon)  Galantamine (Razadyne)  Memantine (Namenda)  Donepezil and memantine (Namzaric) | | | |
